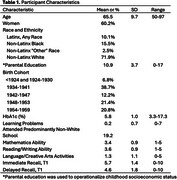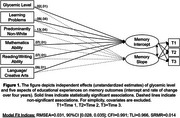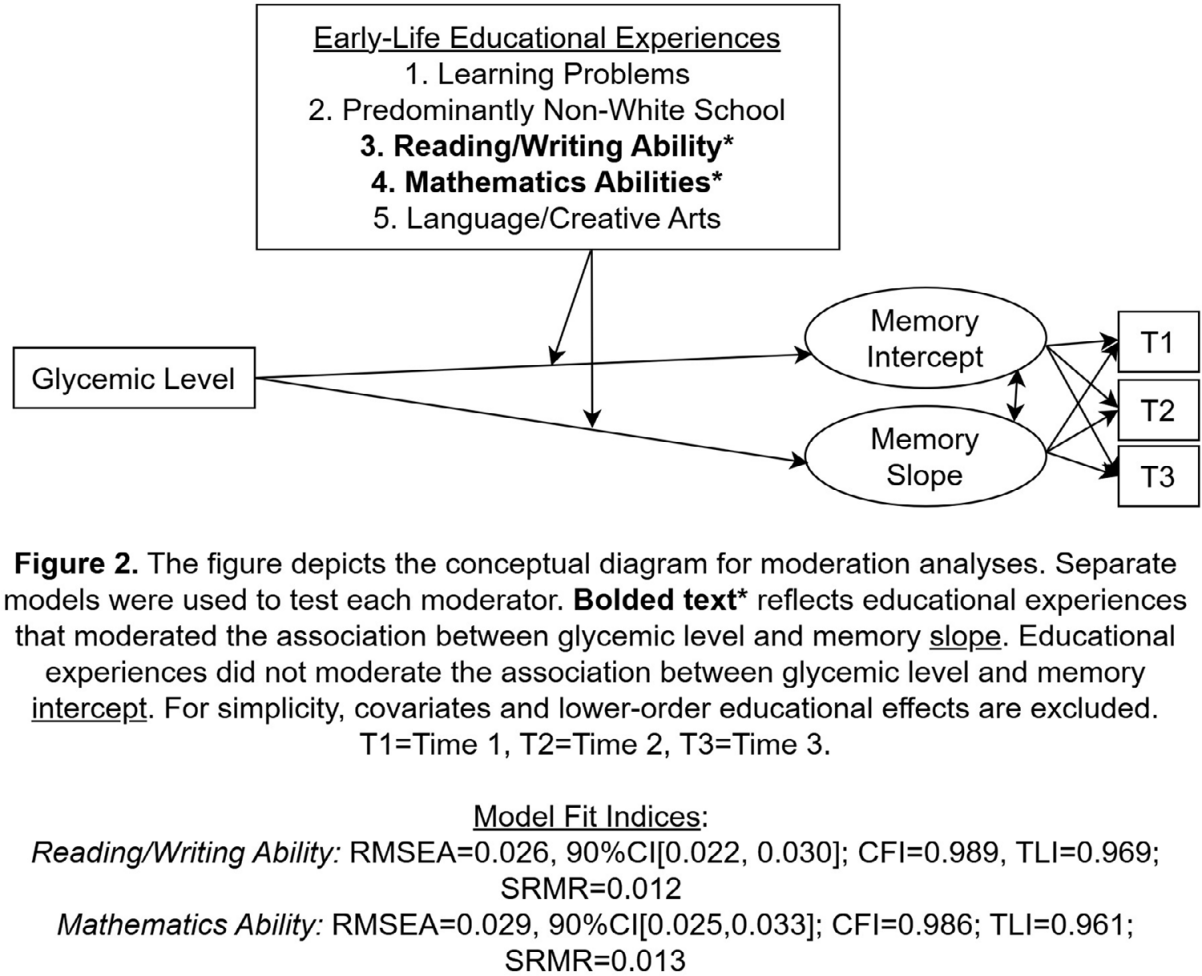# Early‐Life Educational Experiences Moderates the Association Later‐Life Glycemic Level and Memory Trajectories in Later‐Life

**DOI:** 10.1002/alz70860_107253

**Published:** 2025-12-23

**Authors:** A. Zarina Kraal, Jacqui Smith, Jennifer J. Manly

**Affiliations:** ^1^ Taub Institute for Research on Alzheimer's Disease and the Aging Brain, New York, NY, USA; ^2^ Columbia University Irving Medical Center, New York, NY, USA; ^3^ Institute for Social Research, University of Michigan, Ann Arbor, MI, USA

## Abstract

**Background:**

Higher glycemic levels are associated with poor cognitive outcomes in older adulthood. Early‐life educational experiences predict later‐life cognition, but their role as a moderator of the association between hyperglycemia and cognition is unknown. Educational experiences in childhood promotes cognitive reserve, and greater cognitive reserve may reduce the deleterious effects of hyperglycemia on cognition in later‐life. We hypothesized that higher glycemic level and poorer childhood educational experiences would be associated with worse memory outcomes in later‐life, and that the negative association between glycemic level and memory would be stronger in individuals with worse educational experiences.

**Methods:**

Participants comprised 8,142 adults from the Health and Retirement Study Life History Mail Survey (LHMS) with biomarker data (Table 1). Memory was assessed biennially (three timepoints) with immediate and delayed word recall. Glycemic level was indexed by hemoglobin A1c (HbA1c). Retrospectively reported childhood educational experiences included: learning problems, school context (predominantly white vs. non‐white students), mathematics ability, reading/writing ability, and participation in extracurricular language/creative arts activities. Covariates were baseline age, sex/gender, race, ethnicity, birth cohort, parental education, wealth, and assistance with LHMS questionnaire completion.

**Results:**

Latent growth curve models showed independent associations of HbA1c and all education variables with memory intercept, but not memory decline (Figure 1). Models separated by each education variable showed that the association between higher HbA1c and steeper memory decline was stronger in older adults who reported very low reading/writing (*B* = .19, SE=0.10, *p* = .04) and mathematics (*B* = ‐.22, SE=.11, *p* = .04) abilities (Figure 2). Patterns of association were stronger when HbA1c was restricted to non‐normal (≥5.7%) and clinically elevated (≥6.5%) levels. Educational experiences did not moderate HbA1c associations with memory intercept.

**Conclusion:**

The current study provides preliminary evidence suggesting that early‐life educational quality, particularly in mathematics and literacy, may shape hyperglycemia‐related associations with memory aging. Poor educational experiences during formative years may hinder the development of cognitive reserve, exacerbating the adverse cognitive consequences of hyperglycemia. Future studies are needed to establish causal relationships, examine educational experiences beyond high school, and model memory trajectories over a longer follow‐up period, to more fully characterize the contribution of early‐life educational experiences to cognitive health in later‐life.